# Expression and Clinical Significance of Mucin Gene in Chronic Rhinosinusitis

**DOI:** 10.1007/s11882-020-00958-w

**Published:** 2020-08-18

**Authors:** Jiaxin Tong, Qingjia Gu

**Affiliations:** Department of Otorhinolaryngology Head and Neck Surgery, Sichuan Provinicial People’s Hospital & Affiliated Hospital of University of Electronic Science and Technology of China, Chengdu, 610072 Sichuan China

**Keywords:** Chronic rhinosinusitis, Mucin, Corticosteroids, Human neutrophil elastase, Transforming growth factor-β1

## Abstract

**Purpose of Review:**

This review highlights the expression and regulation of mucin in CRS and discusses its clinical implications.

**Recent Findings:**

Chronic rhinosinusitis (CRS) is common chronic nasal disease; one of its main manifestations and important features is mucus overproduction. Mucin is the major component of mucus and plays a critical role in the pathophysiological changes in CRS. The phenotype of CRS affects the expression of various mucins, especially in nasal polyps (NP). Corticosteroids(CS), human neutrophil elastase (HNE), and transforming growth factor-β1 (TGF-β1) are closely related to the tissue remodeling of CRS and regulate mucin expression, mainly MUC1, MUC4, MUC5AC, and MUC5B. “It is expected that CS, HNE and TGF - β could be used to regulate the expression of mucin in CRS.” However, at present, the research on mucin is mainly focused on mucin 5AC and mucin 5B, which is bad for finding new therapeutic targets.

**Summary:**

Investigating the expression and location of mucin in nasal mucosa and understanding the role of various inflammatory factors in mucin expression are helpful to figure out regulatory mechanisms of airway mucin hypersecretion. It is of great significance for the treatment of CRS.

## Introduction

CRS is a clinically common otolaryngology disease, which is prevalent all over the world [1]. CRS is classified into two phenotypes [2, 3••, 4••], based on the tissue remodeling characteristics, referred to as chronic rhinosinusitis with nasal polyps (CRSwNP), and chronic rhinosinusitis without nasal polyps (CRSsNP), respectively [5]. CRS can occur in any age group and the morbidity rate increases with age. At present, the morbidity rate of CRS in China is 2 ~ 8% [6•, 7], as well as the number of CRS patients increases by 0.3% every year [8]. However, the pathogenesis of CRS remains unclear so far [9]. Although CRS is rarely fatal, it can cause nasal congestion, purulent rhinorrhea, reduction/loss of smell, facial pressure or pain, and mucosal edema [10–13]. The symptoms may continue for 12 weeks or more, which bring about a substantial burden in terms of health, quality of life, and economical expenditure [14, 15].

Studies have shown that mucin is the major component of airway mucus in patients with CRS, which affects the rheological properties of mucus [16], leading to a series of pathophysiological changes, including submucosal gland hyperplasia, the increased numbers and excessive secretion of airway goblet cells, and mucin hypersecretion, particularly MUC5AC and MUC5B [17]. MUC5AC and MUC5B, as important components of respiratory secretions, are increased in CRS [18]. Among them, the studies find that MUC5AC plays a critical role in the inflammatory response of the respiratory tract [19], and many pro-inflammatory cytokines regulate goblet cell metaplasia and excessive secretion of MUC5AC [17].

It is speculated that uncontrolled inflammation is responsible for many of the manifestations and symptoms of CRS [20], which is closely related to mechanism of pathogenesis of CRS [21]. There exists some lack of recognition about the pathogenesis of CRS among the circles of medicine presently. Most scholars believed that hyperplasia and metaplasia of glandular cells and goblet cells and motivating expression of mucin are three important pathogenic mechanisms of CRS. However, the mechanism is not well established and remains controversial [22], which still requires more investigation. Nowadays, more and more studies have focused on tissue remodeling in CRS and have indicated that CRS is also distinguished by mucosal remodeling and different subtypes of CRS exhibit different characteristics of tissue remodeling. Currently, the evidence suggests that there is close relationship between inflammation and remodeling [5, 23]. And lots of inflammatory mediators play an important role in this relationship [24, 25]. Therefore, the treatment of CRS is extremely challenging.

## Structure of Mucins

Mucin is the major macromolecular component of airway mucus [26] and exists in the form of high-density glycosylated molecules with molecular weight ranging from 1 to 50,106 kDa [16].Goblet cells in the superficial epithelium cells and submucosal glands (SMG) can rapidly produce mucus under certain stimulation in the form of exocytosis and then form a mucus layer in the airways [14]. The mucus layer is divided into two layers: one layer is the inner serous layer called the sol phase, in which the cilia recover from its active tempo, and the other layer is the outer more viscous layer called the gel phase, in which cilia plays a transport function through pulsation.

To date, 20 kinds of mucins have been identified and can be divided into two broad categories: membrane-bound mucins and secreted mucins [27]. Among them, MUC2, MUC6, MUC8, MUC5AC, MUC5B, and possibly MUC19 are secreted gel-forming mucins, whereas MUC1, MUC3A, MUC3B, MUC4, MUC11 to MUC13, MUC15 to MUC 18, and MUC 20 are membrane-bound mucins [14]. Among these mucins, MUC5AC and MUC5B are the major secreted gel-forming mucins. MUC2, MUC5AC, MUC5B, and MUC6 are present in tandem on the conserved cluster of human chromosome 11p15 and may occur by gene duplication [28]; MUC19 is found on human chromosome 12q12 [29]; MUC3A, MUC3B, MUC11, MUC12, and MUC17 are both mapped to human chromosome 7q22. Among them, MUC5AC, approximately 586 kDa, is a major secreted mucin of the polypeptide chain, which has a large number of O-glycosylated chains linked to Ser and Thr residues in the VNTR regions [30].

**Fig. 1:**
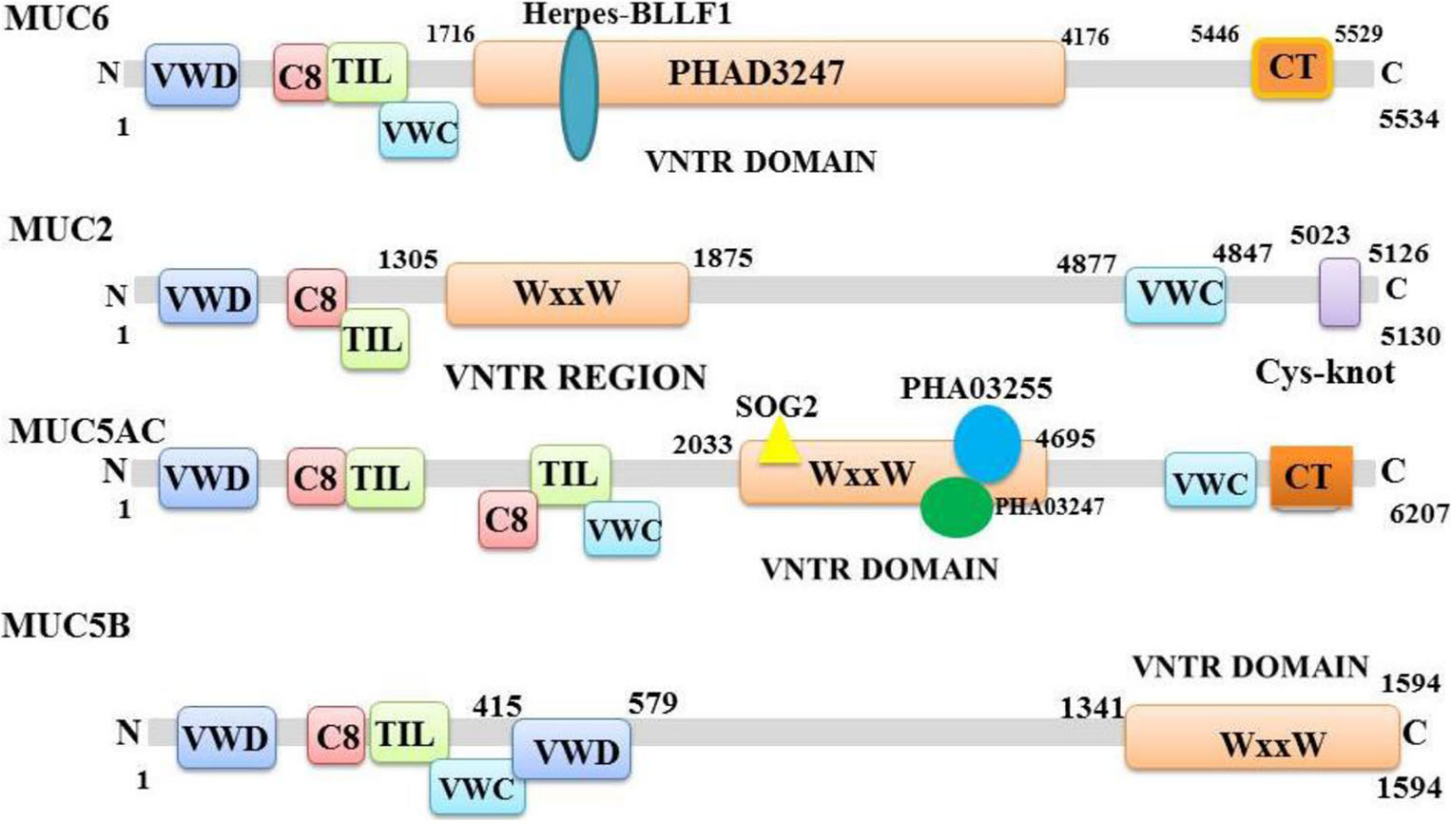
Diagrammatic presentation of conserved domains of MUC2, MUC6, MUC5AC, and MUC5B. The secondary structures of mucins vary greatly in length, number, domain, and VNTR (variable number tandem repeat) domain. MUC2, MUC5AC, and MUC5B all have a common structure as MUC2 protein, namely, the WxxW repeating region, which is a repeating region found in MUC2 and MUC5AC. But the function of this region is unknown and the repetitive sequence can be present in up to 32 copies. The region has a highly conserved WxxW sequence motif and at least six well-conserved cysteine residues. SOG2 protein super family participates in RAM signaling pathway within the scope of MUC5AC protein WxxW repeating region. And there are PHA03255 super family and PHA03247 super family within the scope of MUC5AC protein WxxW repeating region. The PHA03247 super family is in the Herpes-BLLF1 domain in MUC6. (VWD, von Willebrand factor (vWF) type D domain; WxxW, WxxW repeating region; C8, This domain contains 8 conserved cysteine residues; TIL, trypsin inhibitor-like cysteine rich domain; VWC, von Willebrand factor type C domain; Cy-Knot, cystine-knot domain; CT, C-terminal cystine knot-like domain (CTCK))

**Fig. 2:**
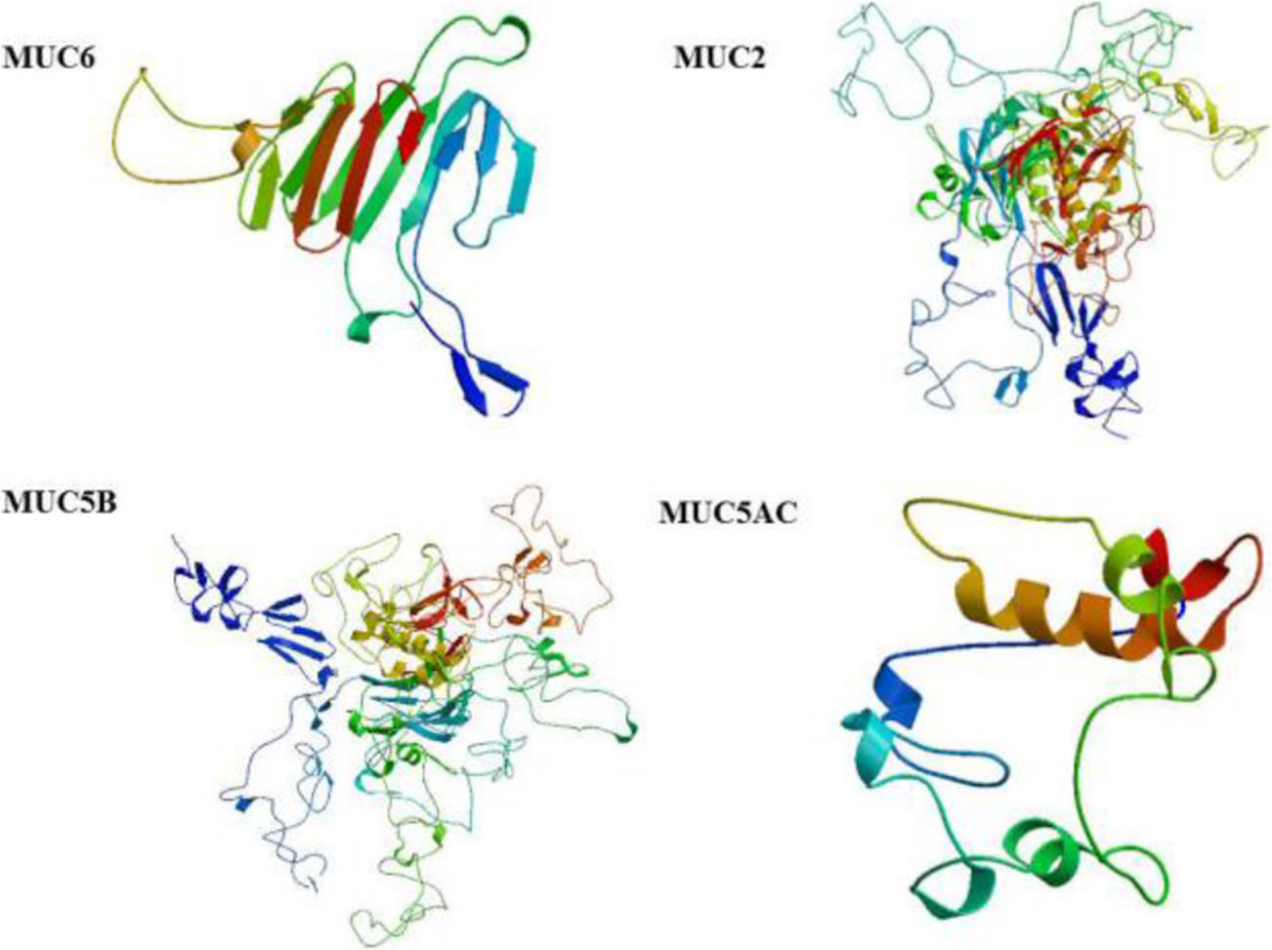
Diagrammatic presentation of tertiary structures of MUC2, MUC6, MUC5AC, and MUC5B. These mucins have specific domains and sequence and size of these domains are unique

The currently accepted molecular model of mucin is a linear and flexible amino acid chain, which is composed of interconnected subunits by disulfide bonds and each subunit contains alternating highly glycosylated proteinase-resistant regions and sparsely non-glycosylated proteinase-sensitive region (Fig. 1). The greatest feature of molecular model of mucin is the variable number of tandem repeats amino acid sequences in the highly glycosylated region, and the number of amino acids varies between 8 and 169. These tandem repeats are rich in serine or threonine, which represent the potential sites for O-glycosylation. In a single mucin gene, the tandem repeat domains exhibit a change in the number of tandem repeat sequence due to genetic polymorphism, which results in a difference in the size of the mucin molecules. Different types of mucins have their own representative characteristics. Membrane-tethered mucins are transmembrane proteins and are anchored to the apical surface of mucosal epithelial cells. Membrane-bound mucins have at least three common features, which include a transmembrane domain, a highly glycosylated N-terminal domain in contact with the outside environment acting as a sensor receptor, and a short cytoplasmic tail (CT) that enables its participation in intracellular signaling [31]. Secreted mucins have at least five major cysteine-rich domains (Fig. 2).

## Expression and Role of Mucins in CRS

In recent years, domestic and foreign scholars have done a lot of research on the secretion, expression, and distribution position of mucin in nasal mucosa. Seventeen mucins have been identified in the lower airways and 8 mucin genes have been reported in the upper airways. And more mucin genes are expected to be identified in future studies. Although various studies have investigated hyperecretion of 20 mucins [32, 33], the expression levels and distribution positions of several mucins are still unclear, such as MUC6, MUC19, and MUC7 (Table 1). The evidence is not clear whether the diagnosis of nasal polyposis is associated with alterations of mucin expression.

**Table 1:** Mucus type, mucin, main tissue expression of 8 mucin genes

Gene	Mucus type	Expression (NP)	Regulation	Chromosomal locus
MUC1	Transmembrane	↑ upregulated	CS	1q21
MUC2	Secreted	↑ upregulated		11p15.4–11p15.5
MUC3	Transmembrane	↓ downregulated		
MUC4	Transmembrane	↑ upregulated	CS	3q29
MUC5AC	Secreted	↑ upregulated	CS, TGF-β1, HNE	11p15.5
MUC5B	Secreted	↑ upregulated	CS, HNE	11p15.5
MUC6	Secreted	↓ downregulated		11p15.5
MUC7	Secreted-non-polymeric			4q13.3
MUC8	Secreted-non-polymeric	↑ upregulated		12q24.3

Aust et al. [34] and Kim et al. [35] reported the expression of MUC1, MUC2, MUC4, MUC5AC, MUC5B, MUC7, and MUC8 in normal nasal mucosa. Quantitative changes and localization changes in expression of mucin were investigated by immunohistochemical analysis. The results showed that MUC5AC was only identified in surface epithelial goblet cells and MUC5B was only expressed at low levels in nasal sinus SMG. Quantitative analysis of mucin secretion in CRS by ELISA indicated that most mucins are derived from SMG; however, this method failed to identify which kind of mucin. The results of Aust et al. [34] showed that the expression of MUC3 and MUC6 was weakened in nasal mucosa and nasal polyps. The results of Kim et al. [35] demonstrated that MUC3 and MUC6 were excluded from the study of expression of MUC1 to MUC8 in nasal polyps. All studies showed that MUC2 and MUC8 were more strongly expressed in nasal polyps than nasal mucosa. Martínez et al. [36] found that the expression levels of MUC5AC mRNA and MUC5B mRNA in CRS were significantly increased in CRS compared to in normal sinus mucosa. Studies on the expression of mucin in nasal polyps by using probes to be directed against unique sequence of mucin molecule (discontinuous repeat probes) show that expression level of MUC5AC is four times higher than MUC2 and is twelve times higher than MUC1. Using in situ hybridization, the researchers compared the expression of 8 mucins (MUC1, MUC2, MUC3, MUC4, MUC5AC, MUC5B, MUC6, MUC7, and MUC8) in nasal polyps and normal sphenoid sinus mucosa. The results showed that the location of 8 mucins is in nasal polyps, which demonstrated that more mucin genes were activated during the development of nasal polyps. We find that the major variations of expression of mucin genes in nasal polyps have also been reported to have similar results in CRS. This suggests that the most significant variations of expression of mucin genes involve SMG rather than epithelium with the development of nasal polyps [16]. Lee et al. [37] used reverse transcription polymerase chain reaction and immunohistochemistry to detect the expression and distribution of MUC8 in the maxillary sinus mucosa in CRS. The results showed that the expression intensity and distribution density of MUC8 increase obviously. Jung et al. [38] examined the expression of mucin in the ethmoid mucosa of 8 patients with CRS. The results revealed that there were 2 cases of MUC1 expression, 6 cases of MUC4 expression, 8 cases of MUC5AC expression, 5 cases of MUC5B expression, 7 cases of MUC7 expression, and 8 cases of MUC8 expression. However, MUC2 and MUC6 were not detected, which proved that MUC4, MUC5AC, MUC5B, MUC7, and MUC8 were the major mucins in the ethmoid mucosa. Some studies investigated the pattern of expression of mucin in healthy nasal tissues and nasal polyps, which found that overexpressions of MUC1, MUC4, MUC5AC, and MUC5B are in nasal polyps than in healthy nasal tissues [39]. Groneberg et al. [40] have confirmed that MUC5B is expressed in goblet cells and SMG in normal nasal mucosal epithelium, and yet MUC5AC is expressed in goblet cells in normal nasal mucosal epithelium. Sharma et al. [41] fully demonstrated that MUC5B is expressed in mucous cells, while MUC7 was only expressed in the serous cells of the submucosal glands of the trachea.

Under normal circumstances, the mucous glands of the nasal mucosa and the goblet cells in the epithelium secrete mucus, which can keep the nasal mucosa moist, maintain nasal physiological function, and prevent lesions. The goblet cells proliferate and differentiate to maintain their balance of quantity in the nose and sinus. When the nasal mucosa has chronic inflammation, the mucous glands of the mucous and the goblet cells secret excessive mucus, which affect the mucociliary clearance rate [16, 42], leading to mucus retention, aggravating inflammation, and forming a vicious circle, which can also lead to respiratory complications [43].

According to the results of pathological observation [44], mucosal edema, thickening of basement membrane, fibroblast proliferation, collagen deposition, and connective tissue increasing are common remodeling forms of CRS [5], which seriously affect normal sinonasal physiological function [45]. The two phenotypes of CRS [3••, 4••] have different immunoregulatory mechanisms and remodeling features. CRSwNP is a Th2-skewed response with high levels of IL-4, IL-5, and IL-13 [46], which is characterized by albumin deposition, stromal edema, and fibrosis. Conversely, CRSsNP is Th1-skewed response with high levels of IFN-γ; TGF-β1 [47], which is characterized by goblet cell hyperplasia [48]; fibrosis; excessive collagen deposition; and thickening of basement membrane [24, 49]. In short, CRSsNP shows neutrophil infiltration, whereas CRSwNP shows eosinophil predominance, whose symptom is known to be more severe than CRSsNP. Thus, the recurrence rate of nasal polyps is still very high even after surgical removal [50].

Tissue remodeling has been known to be associated with glandular hyperplasia, inflammatory cell infiltration, and mucosal fibrosis [51••, 52]. Therefore, tissue remodeling is considered to be the main influencing factor of CRS [53]. Tissue remodeling is the process of rebuilding an existing tissue that cures the wound by secreting the extracellular matrix when the tissue is damaged [54]. Many inflammatory factors may be involved in tissue remodeling; for example, transforming growth factor-β1 regulates tissue remodeling and induces myofibroblastic differentiation [55]. Activation of fibroblasts produces myofibroblasts and induces extracellular matrix deposition and remodeling [56]. Therefore, fibroblasts can be used as an important target cell to treat CRS. In principle, CRS is regulated by inhibiting ECM accumulation, preventing nasal fibroblastic differentiation, and tissue remodeling, but whether it can be clinically achieved requires further confirmation [57].

## Regulation of Mucins in CRS

The researchers found that respiratory pathogens and inflammatory cytokines regulate the expression of airway mucin [24, 25]. In NP-derived epithelial cells, pro-inflammatory cytokines such as TNF, IL-4, IL-13, IL-8, and IL-17A can upregulate the transcription and protein level of MUC5AC. Similarly, TNF, TGF-b, IFC-c, and IL-1B upregulate MUC5AC transcription and protein levels in normal sinusoidal epithelial cells treated with inflammatory mediators and pro-inflammatory cytokines such as neutrophil elastase, IL-4, IL-9, and IL-13. In contrast, in normal nasal epithelial cells, pro-inflammatory cytokines including TNF, IL-1B, LPS, IL-4, and PAF all downregulated the expression of MUC5AC [58]. The study demonstrates that a short course of oral steroids increases membrane-tethered (MUC1 and MUC4) mucins and that long-term intranasal steroid treatment is able to decrease major secreted mucins (MUC5AC and MUC5B). The downregulation of secreted mucins could result from the ability of CSs to reduce GCH and could account for the reduction of mucus production and rhinorrhea [59].

The histological features of sinus mucosal lesions in CRS are the large number of inflammatory cell infiltration and cytokine release [60], particularly neutrophils [52, 61, 62], which were reported to be associated with mucosal remodeling. Sampson et al. [63] showed that neutrophils played a role in remodeling by secreting recombinant mediators (such as MMPs and TGF-β) [64]. Lou et al. [65] observed inflammatory cell infiltration status and then used the cluster analysis method to classify the cytological phenotype of CRSwNP and found that the neutrophil type cells accounted for 8%. Neutrophils regulate the excessive secretion of mucin through corresponding signaling pathways (such as tumor necrosis factor, α-convertase, TGF-β, and epidermal growth factor receptor). The indirect effects of pendrin protein on the recruitment of inflammatory cells (such as neutrophils) may also induce excessive production of mucin. In Chinese patients with CRS, the pendrin protein may accelerate the excessive secretion of MUC5AC by promoting the neutrophil infiltration and goblet cell proliferation. The pendrin is an anion-transporting protein and plays an important role in mucus production. However, the specific pathological mechanism of increasing MUC5AC secretion by pendrin protein is still unclear, which still needs further research.

**Fig. 3:**
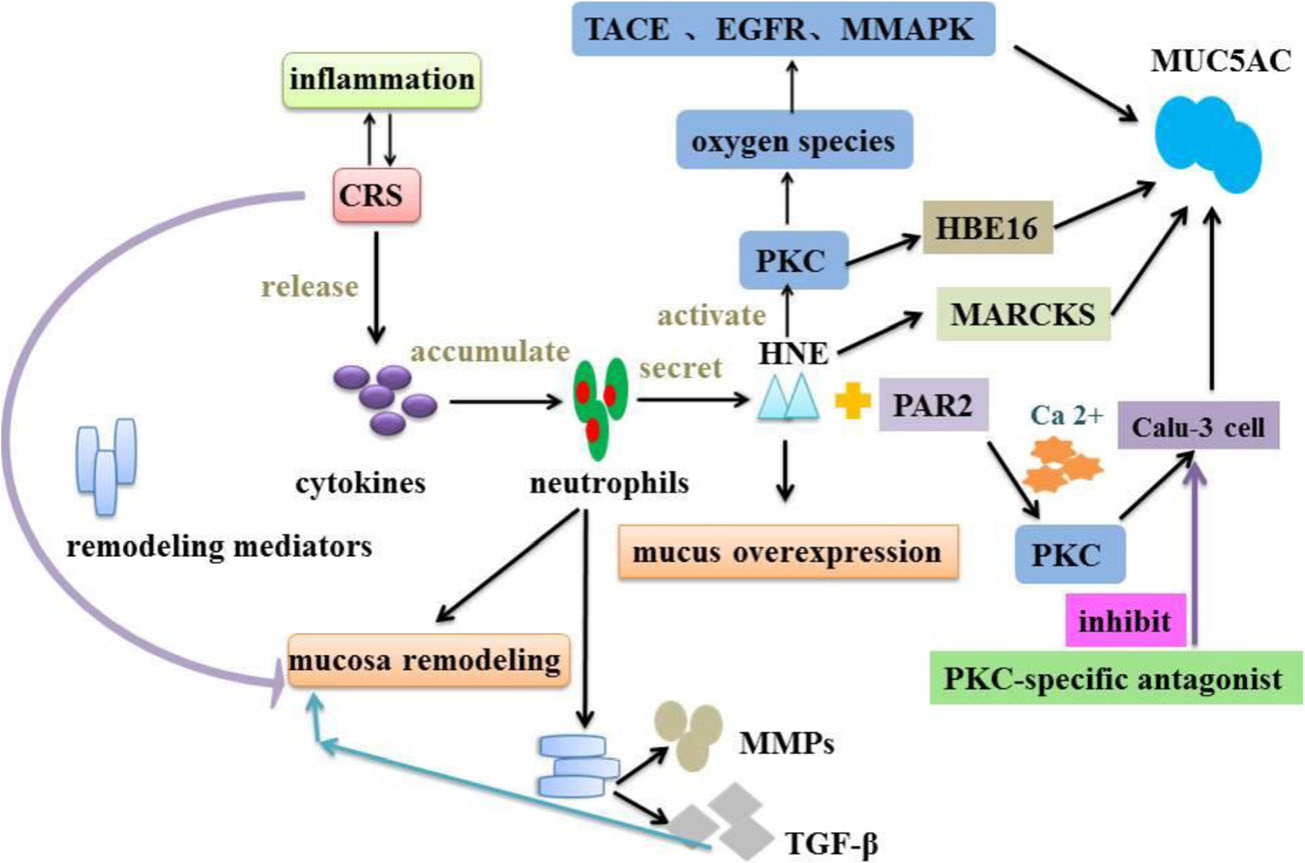
HNE regulates MUC5AC secretion. HNE induces MUC5AC overexpression by several signaling pathways. HNE can activate PKC to produce reactive oxygen species and then activate TACE, EGFR, and MAPK, which ultimately result in mucin production. HNE also induces MUC5AC secretion in HBE16 cells via activating PKC. In addition, HNE can make MARCKS phosphorylation to activate MUC5AC exocytosis. HNE and PAR2 can activate PKC via increasing Ca^2+^ concentration and PKC induces MUC5AC secretion in Calu-3 cells. However, a PKC-specific antagonist can inhibit MUC5AC exocytosis in Calu-3 cells. (PKC: protein kinase C; PAR2, protease activated receptor 2; TACE, TNF-α converting enzyme; EGFR: epidermal growth factor receptor; MARCKS, myristoylatde alanine-rich C kinase substrate)

HNE is a serine protease [66] and released by neutrophil degranulation [67], forming the neutrophil inflammatory mediator [68] and inducing mucin overproduction and goblet cells metaplasia [69]. Many studies have showed that there is a link between CRS and inflammatory granulosa proteins, such as human neutrophil elastase (HNE) [70] (Fig. 3).

HNE has a significant effect on the immune status of the sinus and the entire airway mucosa. Studies have shown that HNE induces the secretion of MUC5AC and MUC5B [71] and the expression of MUC1, MUC4, and MUC5AC in extracorporeal epithelial cells [72–75]. In addition, other studies have indicated that HNE can cause airway goblet cell hyperplasia. In vitro and in vivo experiments of airway epithelial cells revealed that goblet cells can metabolize and proliferate and increase MUC5AC expression and secretion [69, 76]. Voynow and her colleagues [77] proved that HNE induced goblet cell metaplasia in the airway [78] and resulted in mucin overproduction [79]. Further studies [80, 81] have showed that HNE could increase MUC5AC expression in airway epithelial cells via tumor necrosis factor-converting enzyme (TACE) in the respiratory tract [82, 83]. Recent clinical observational studies also suggested that HNE is a key risk factor for the onset and persistence of bronchiectasis [84, 85]. Seshadri et al. [86] show that similar results in patients with CRS and nasal polyp. Although many reported studies showed that HNE and TACE can induce goblet cell proliferation in lower respiratory tract diseases, the relationship is still unclear in CRS [87].

**Fig. 4:**
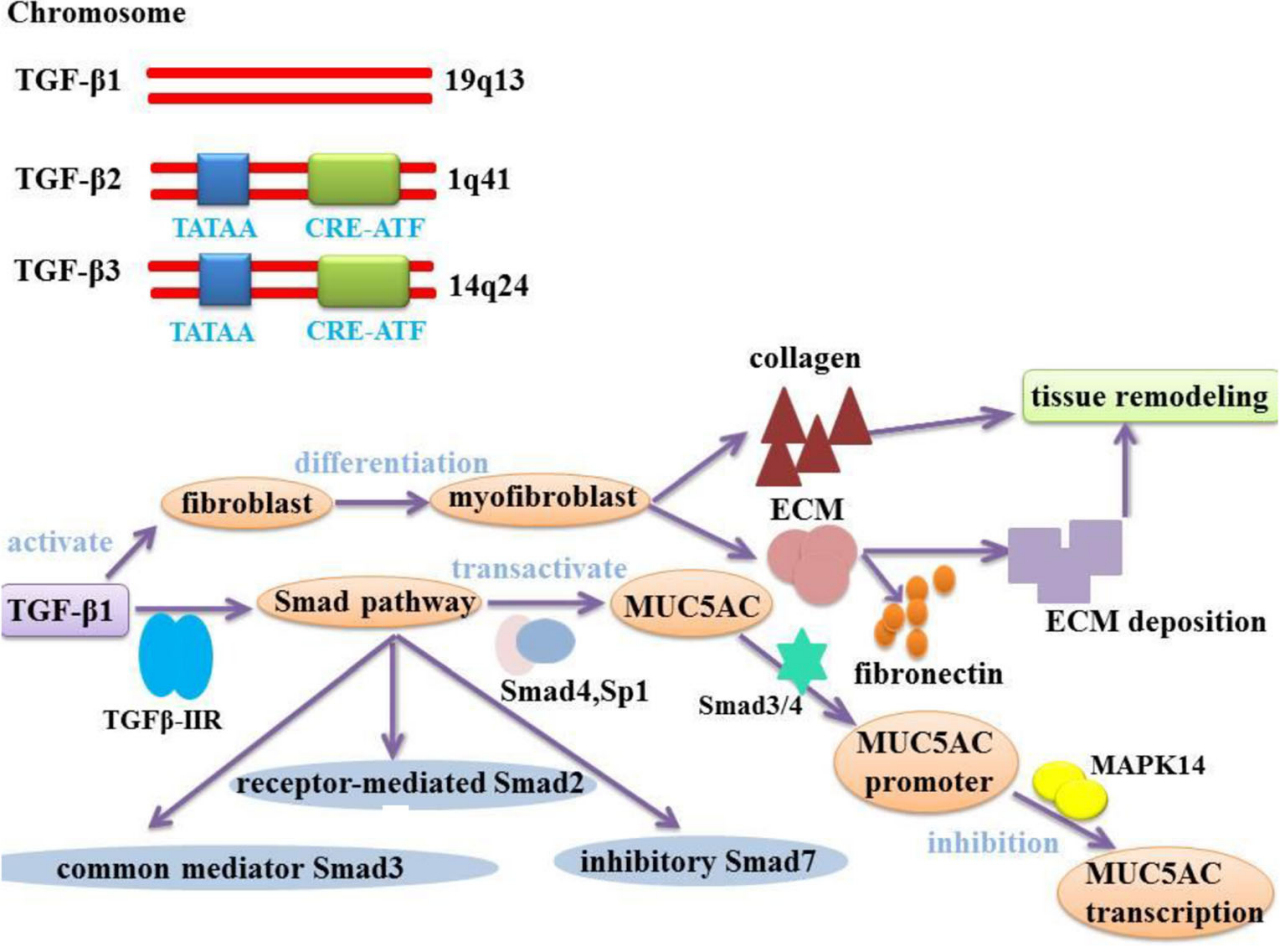
TGF-β participates in the regulation of airway inflammation and remodeling process. Three TGF-β isoforms have been found in the human body, respectively: TGF-β1, TGF-β2, TGF-β3.TGF-β1, TGF-β2, and TGF-β3 are localized on chromosomes 19q13, 1q41 and 14q24, respectively. A promoter for TGF-β2 and TGF-β3 isoforms possesses in its structure a classical TATAA box domain and a CRE-ATF terminal region. TGF-β1 stimulates fibroblast activation and promotes the differentiation of fibroblasts into myofibroblasts and accelerates collagen synthesis in tissue remodeling. Differentiated myofibroblasts increased the expression of ECM components such as fibronectin, promoting massive amounts of ECM deposition, which leads to tissue remodeling. In addition, TGF-β1 binds to receptor TGFβ-IIR and activates Smad signal transduction pathway. TGF-β1 is an important factor in the transactivate MUC5AC promoter activity through Smad4 and Sp1.TGF-β1-Smad3/4 signaling acts as a negative regulator via MAPK14. Inhibitor in MUC5AC transcription. (MAPK14:Mitogen-activated protein kinase 14; Sp1:specificity protein 1)

TGF-β (transforming growth factor beta) is a multifunctional cytokine with important immunomodulatory and fibroblastic properties. TGF-β regulates the inflammation and remodeling of CRS [88, 89], which can produce in the airway inflammatory cells and permeate in the bronchial mucosa [90]. Although nowadays five TGF-β subtypes have been identified, only three subtypes are found in the human body including TGF-β1, TGF-β2, and TGF-β3 [91]. TGF-β1, TGF-β2, and TGF-β3 are localized on chromosomes TGF-β1-19q13, TGF-β2-1q41, and TGF-β3-14q24, respectively. The regulations occur on the transcriptional level, but the function is not entirely known. The promoters of TGF-β2 and TGF-β3 have a classical TATAA box domain and a hormone-controlled CRE-ATF terminal domain in their structure [92] (Fig. 4). The study identified that TGF-β1 and TGF-β2 genes were regulated by miR-532-3p and miR-500a-5p, respectively [93]. It has been reported that TGF-β from regulatory T cells (Tregs) is related to Tregs production and inhibition function of CD4T cells [93].The decrease of TGF-β 1 expression and TGF-β receptor 2 in CRSwNP results in the absence of Tregs in CRSwNP. TGF-β 1 not only affects Tregs but also destroys the integrity of Tregs in CRSwNP, which partly explains the defect of epithelial barrier in CRSwNP [93]. This evidence suggests that TGF-β is closely related to the pathogenesis of CRSwNP. Here, the role of miR-532-3p and miR-500a-5p will be the targets to further understand the role of TGF-β in CRSwNP [89, 94, 95].

According to previous reports, TGF-β1 is a representative profibrotic cytokine and a major stimulator of fibroblast activation, which can induce activation and differentiation of fibroblasts into myofibroblasts. Activated fibroblast or myofibroblast has collagen contractile activities and can initiate tissue remodeling [96]. Therefore, TGF-β1 is closely related to the pathogenesis of CRS. TGF-β1 stimulates the differentiation of fibroblasts into myofibroblasts. The differentiated myofibroblasts increase the expression of ECM components (such as fibronectin and collagen type I), which can promote abundant ECM deposition, leading to airway tissue remodeling [97, 98]. TGF-β is known to activate both Smad-dependent and Smad-independent pathways after binding to its receptor. In miR-32-3p, miR-548e-3p, and miR-3149 miRNAs, miR-548e-3p is the only miRNA involved in the regulation of Smad2, Smad4, and MAPK1 genes in TGF-β signaling pathways. To sum up, miR-532-3p, miR-500a5p, and miR-548e-3p are the three most important miRNAs for the study of TGF-β signaling pathway in CRSwNP94–96.It binds to TGF-β-IIR receptor and activates Smad pathway [99]. TGF-β1 signal transduction is regulated primarily by the Smad proteins: receptor-mediated Smad2, common mediator Smad3, and inhibitory mediator Smad724. TGF-β1 is considered to be an important factor for MUC5AC transactivation promoter activity in a synergistic manner through the Smad4 and Sp1 pathways [100]. However, studies have shown that MAPK14 phosphatase-1-dependent inhibitor can act as a negative regulator of MUC5AC transcription under the TGF-β1-Smad3/4 signal pathway [101]. In addition, the effect of TGF-β2 subtype on MUC5AC expression is also controversial. Previous studies have demonstrated that TGF-β2 could result in a decrease in the expression of both MUC5AC and MUC5B in human bronchial epithelial cells. TGF-β2 can also partially reduce IL-13-induced MUC5AC production by binding to MUC5AC promoter alone in the Smad4 signaling pathway [102]. Another study suggested that IL-13 could induce TGF-β2 expression in vitro and TGF-β2 can promote mucin expression in airway epithelial cells [101]. However, there is no direct and unequivocal evidence that whether TGF-β3 regulates MUC5AC expression, which needs further investigation.

Imbalance of TGF-β subtype activation and expression suggests that TGF-β participated in regulating airway inflammation and remodeling [103], especially regulating mucus hypersecretion in the airway epithelial cells [102]. New evidence suggested that TGF-β can enhance collagen synthesis during tissue remodeling process [24]. Clinical studies [104] also showed that patients with CRSsNP usually have higher expression levels of TGF-β compared with healthy individuals, whereas patients with CRSwNP have lower expression levels of TGF-β. Moreover, Nicholas et al. [53] demonstrated that the decrease of expression levels of TGF-β1 may be associated to the edema formation in CRSwNP, whereas increase of expression levels of TGF-β1 may play a critical role in the excessive tissue repair and fibrosis formation in CRSsNP. As reported by the previous study, TGF-β1 concentration, mRNA expression, and the number of activated Smad2-positive cells (the indication of TGF-βactivation) are significantly higher in patients with CRSsNP than those in healthy individuals. In contrast, in patients with CRSwNP, this phenomenon was not observed [53, 105]. At the same time, TGF-β1 may be affected by many factors in CRSwNP and further research is needed to confirm this hypothesis.

Corticosteroids (CS) are the first-line treatment drug for NP, which has strong anti-inflammatory activity [106] and can reduce its volume and inflammatory component. However, its effect on mucin hypersecretion has been controversial. Corticosteroids play an anti-inflammatory role by binding to the intracellular receptor the glucocorticoid receptor (GR), which is a ligand induced transcription factor [106]. In ligand binding, GR complex is transferred to the nucleus with the help of a number of proteins, such as nuclear localization signals and input protein, and exerts its anti-inflammatory effects [107]. There are two ways of action of CS: one is to directly regulate the expression of mucin [108]; the other is to indirectly inhibit pro-inflammatory cytokines [109]. Milara et al. suggested that downregulation of MUC1 in NP tissues is related with anti-corticosteroid in CRSwNP [106]. In contrast, MUC4 was significantly overexpressed in NP epithelial cells of corticosteroid resistant patients when compared with CRSwNP responder patients [110]. Further analysis showed that the cytoplasmic tail (CT) part of MUC1 has anti-inflammatory effects on nasal polyp epithelial cells by inhibiting toll-like receptors (TLR) [31, 111, 112].. In addition, the formation of MUC1-CT and glucocorticoid receptor alpha (GRα) protein complex can protect GR-Ser226 hyperphosphorylation induced by TLR agonists and help to mediate GRα nuclear translocation in response to corticosteroids, so as to play an anti-inflammatory role [106]. Recent evidence suggests that after 2 weeks of oral administration of corticosteroids, corticosteroids increase MUC1 expression in vitro and in human NP epithelium. However, the relationship between the efficacy of oral corticosteroids and the expression of MUC1, as well as the possible interactions among corticosteroids, GR, and MUC1, is not clear [106]. For example, it is not clear whether there is a direct interaction between MUC1-CT and GRα, because MUC1-CT can indirectly bind to the GRα chaperone complex. Recent evidences shows that MUC4 expression is upregulated in airways under inflammatory conditions and that corticosteroids reduce MUC4 expression in vitro [113–115]. However, the relationship between the efficacy of oral corticosteroids and the expression of MUC4, as well as the possible interaction among corticosteroids, glucocorticoid receptor (GR), and MUC4, is not clear [110]. The downregulation caused by CSs in MUC5AC and MUC5B levels may lead to the decrease of mucus hypersecretion in NP. In this direction, the downregulation of MUC5AC after CS treatment was significantly related to the improvement of nasal urea in all NP patients [59].

## Conclusion

In this review, we provide supporting evidence that the expression and location of mucin in normal nasal mucosa and CRS mucosa are different, depending on the phenotype of CRS, various inflammatory factors, and types of mucin (secretory or membrane binding). Most of the studies focus on the expression of mucin in nasal polyps and normal nose, and only a few studies compare the expression of mucin in normal nose and CRS. Compared with normal nasal mucosa, the expression of MUC3 and MUC6 in nasal polyps is downregulated, the expression of MUC2 and muc8 in nasal polyps is upregulated, the expression of MUC5AC and MUC5B in CRS is upregulated, and the expression of MUC5AC in nasal polyps far exceeded MUC1 and MUC2. At present, only MUC8 is upregulated in maxillary sinus mucosa, MUC2 and MUC6 are not expressed in ethmoid sinus mucosa, and no other mucin is found. According to the existing results, we find that MUC5AC is distributed in epithelial goblet cells and SMG, MUC5B is distributed in SMG, and the distribution of other mucins needed further study. The regulation of mucin depends on various inflammatory factors. In CRSwNP, there are few studies on the downregulation of MUC5AC and MUC5B by CS, which is not enough to draw accurate conclusions and remains to be studied. At present, only HNE upregulated MUC5AC and MUC5B, TGF-β upregulated MUC5AC, and few other mucins were involved. In conclusion, the role of various inflammatory factors in the regulation of mucin expression provides a good direction for clinical treatment.
